# A Study of Concept to Prepare Totally Biosourced Wood Adhesives from Only Soy Protein and Tannin

**DOI:** 10.3390/polym14061150

**Published:** 2022-03-13

**Authors:** Saman Ghahri, Antonio Pizzi, Reza Hajihassani

**Affiliations:** 1 Wood and Forest Products Research Division, Research Institute of Forests and Rangelands, Agricultural Research, Education and Extension Organization (AREEO), Tehran 19395-1113, Iran; reza.hajihassani@gmail.com; 2 LERMAB, Faculte des Sciences, University of Lorraine, Blvd. des Aiguillettes, 54000 Nancy, France

**Keywords:** soy protein isolate, SPI, tannin, co-reaction, covalent cross-linking, wood bio adhesives

## Abstract

This is a study of concept on the initial application for wood adhesives totally biosourced from the covalent reaction between soy protein isolate (SPI) and a commercial flavonoid tannin, namely quebracho tannin. The adhesive is composed exclusively of the two vegetable biomaterials mentioned and thus is totally biosourced and non-toxic, as tannin has been classified as being not at all toxic by the European Commission REACH program. The pre-reaction between the two yielded the best plywood bonding results when limited to a temperature of 40 °C, final cross-linking being achieved during the plywood higher temperature hot pressing procedure, as for any other thermosetting adhesive. Pre-reaction at higher temperatures, namely 60 °C and 80 °C, achieved extensive premature cross-linking that lost any activity to cross-link further when hot pressed for preparing plywood. The reaction was followed by thermomechanical analysis, by matrix assisted laser desorption ionization time of flight (MALDI ToF) mass spectrometry, and by plywood shear strength tested dry, after a 24 h cold water soak and 1 h in boiling water. The adhesive of this approach lends itself to be further reinforced by the multitude of approaches on soy resins already developed by several other research groups.

## 1. Introduction

Soy-based resins, being soy protein and flour biosourced, are now of particular interest, particularly for bonding wood panel adhesives. The literature abounds with approaches adding soy protein or flour to supplement traditional synthetic wood panel adhesives, as well as using partially hydrolyzed or modified soy resins, both presenting good results, for the same application [1,2,3,4,5,6,7,8,9]. Many researchers have already tried to prepare acceptable soy-based resins, but most of the approaches used show some drawbacks. Several different synthetic additives have been used to improve their performance, such as polyamidoamine-epichlorohydrin (PAE), phenol formaldehyde resins, and Na dodecyl sulfate [6,10,11], and other equally interesting approaches as well [12,13]. While this is not really a problem, due to the growing awareness of environment pollution, the focus of the research on soy panel adhesives is shifting instead to using the highest possible percentage of materials of renewable origin and to minimize or even eliminate synthetic, oil-derived chemicals. Vegetable condensed and hydrolysable tannins are one good choice to modify soy adhesives for wood panels, as they are classed as non-toxic, even for human consumption, and are classified as such by the relevant Registration, Evaluation, Authorization, and Restriction of Chemicals (REACH) classification of the European Commission [14]. They are commercially obtained by water extraction from the bark or wood of several tree species [15,16] from several perennial shrubs [15] and also from residual grape skins after grape juice extraction [17].

Recently, research work on the co-reaction of condensed and hydrolysable tannins and soy protein isolate (SPI) has shown the potential of preparing good particleboard and plywood wood panel adhesives [18,19,20,21,22]. This work has evolved even more recently on to more advanced and effective adhesive formulations [23], and consequently with the very recent finding that proteins start and do progressively react more covalently with tannins as the temperature increases, such as at 80 °C [24]. The reaction is predominantly due to the reaction of the protein lateral chains amino groups substituting some of the phenolic hydroxyl groups of the tannin. Tannin amination occurs with ease, is well known, and has already been reported [25,26,27,28]. However, even the most recent research work [21] still used hardeners such as hexamethylenetetramine (hexamine), although to a much reduced level, to make the system work. However, what is really sought is to prepare an adhesive based exclusively on a protein and on tannin, without any hardener of any type, and thus totally biosourced based on the covalent reaction between the two materials at higher temperature. It is for this reason that the present work builds on previous research work for the bonding of interior grade plywood with just SPI and a tannin and nothing else. The two reactants in the present work were then expressly chosen, one for being a vegetable protein (soy protein), and the second a more available commercial condensed tannin extract obtained by hot water extraction from the quebracho tree, but the approach is equally valid for other condensed tannins. The present demonstration of the feasibility of this approach is done to render possible the reinforcing of this system by the superposition to it of other approaches using small additions of other materials, as already reported by a number of different authors [3,4,5,6,8,9,10,11,12,13,18,19,20,21,22,23,24,29].

## 2. Materials and Methods 

### 2.1. Materials

Defatted soy flour (SF) with 47 wt% protein content was purchased from BEHPAK Co. (Behshahr, Mazandaran, Iran). The soy protein isolate (SPI) with 90 wt% protein content used in this study was donated by ADM Co. (Decatur, Illinois, USA). Quebracho wood tannin (*Schinopsis balansae* and *Schinopsis lorentzii*) extract (produced by Indunor Argentina, part of SILVA Chimica, S. Michele Mondovi, Italy) was used.

### 2.2. Adhesive Formulations

Adhesive formulations were described in previous research work [20]. Different amounts of SF (9.38 g) were added to distilled water (23.4 g) and mixed with a mechanical stirrer at room temperature (21 ± 2 °C). The tannin extract (9.38 g) was added to distilled water (13.1 g) and stirred well. The tannin solution was then added to the soy slurry and stirred for 30 min. After preparing the soy–tannin (50% wt/50% wt) adhesive, the mixtures were then preheated each at different temperatures (40 °C, 60 °C, and 80 °C) for 30 min, as reported in previous work [23]. The tannin solutions in water were prepared at 42 wt% concentration. The control sample was prepared at ambient temperature (20 ± 2 °C).

### 2.3. Matrix-Assisted Laser Desorption/Ionization Time-of-Flight (MALDI-ToF) Mass Spectrometry

Quebracho tannin extract was reacted at ambient temperature and at 80 °C for 1 h with soy protein isolates (ISP). The test procedure followed was according to the method previously described by Chen et al. [23] for soy protein and mimosa tannin, and Pizzi [24] for collagen and mimosa tannin and tannin and soy protein [25]. The samples for MALDI-ToF analysis were prepared by first dissolving 7.5 mg of sample powder in 1 mL of a 50:50 *v/v* acetone/water solution. Then 10 mg of this solution was added to 10 mL of a 2,5-dihydroxy benzoic acid (DHB) matrix. The locations dedicated to the samples on the analysis plaque were first covered with 2 mL of a NaCl solution 0.1 M in 2:1 *v/v* methanol/water, and pre-dried. Then 1.5 mL of the sample solution was placed on its dedicated location, and the plaque was dried again. Red phosphorous was to standardize the MALDI ToF equipment. MALDI ToF spectra were obtained using an Axima-Performance mass spectrometer from Shimadzu Biotech (Kratos Analytical Shimadzu Europe Ltd., Manchester, UK) using a linear polarity-positive tuning mode with a gate value of 400 Da.

### 2.4. Thermomechanical Analysis (TMA) 

The pre-reacted SPI–quebracho tannin and for completion soy flour–quebracho tannin mixes in 90:10 weight proportions 90:10 were tested dynamically by thermo-mechanical analysis (TMA) on a Mettler 40 apparatus (Mettler-Toledo, Viroflay, France) according to procedures already developed [17,24,25] with SPI only and soy flour only controls. Different samples composed of two beech wood plies, each 0.5 mm thick, bonded with each adhesive system, for sample dimensions of 17 mm × 5 mm × 1 mm, were tested in non-isothermal mode between 25 °C and 250 °C at a heating rate of 10 °C/min in three-point bending. A force varying continuously between 0.1 N, 0.5 N, and back to 0.1 N was applied on the specimens with each force cycle of 12 s (6 s/6 s). The classical mechanics relation between force and deflection E = [L^3^/(4bh^3^)][DF/(Df)] (where L is the sample length, b and h are the sample width and thickness, DF is the variation of the force applied, and Df is the deflection obtained) allows the calculation of the modulus of elasticity (MOE) E for each case tested. Different formulations of adhesives were tested by TMA [22,30,31].

### 2.5. Plywood Preparation and Testing

Duplicate 3-ply poplar (*Populus nigra*) veneer laboratory plywood panels of dimensions 350 mm × 350 mm × 6 mm were prepared. Glue mix spread was a 300 g/m^2^ double glue line. Pressing conditions were 11 min at 160 °C and 11 kg/cm^2^. After cutting, the specimens were tested in tension dry, after 24 h soaking in cold water, and 1 h soaking in boiling water, tested wet, according to EN 314-1 standard [32].

## 3. Results and Discussion

**Figure 1:**
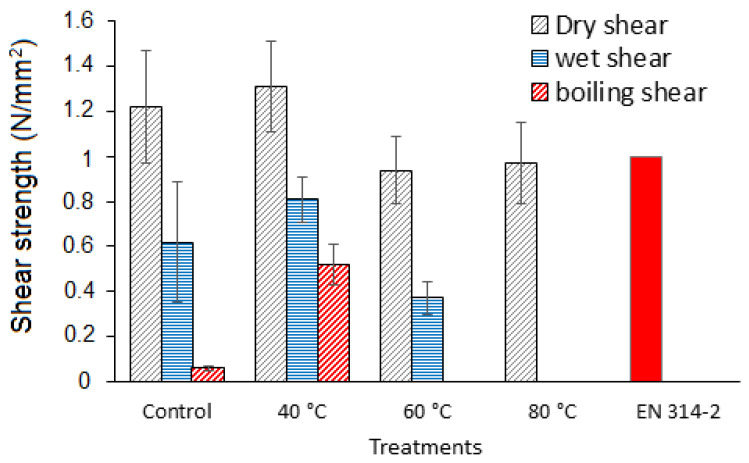
Effect of pre-heating treatment (40, 60, and 80 °C) on shear strength of plywood specimens in different conditions (dry, cold water soaking, and boiling water soaking). Control: Ambient temperature (20 ± 2). EN 314-2 specifies 1 N/mm^2^ for dry shear strength of plywood [32].

The results of the plywood panels shear strength are shown in Figure 1 and are of interest. They show that pretreating the 50:50 SPI–quebracho tannin adhesive at 40 °C gave better results than the SPI alone control, but as preheating passed at higher temperatures, the performance of the bonded plywood progressively decreased. The results also indicated that the 40 °C pretreated SPI–tannin adhesive, while presenting only a moderate improvement in dry strength, presented much better 24 h cold water strength (30% improvement, from 0.62 for the control to 0.81 MPa for the SPI–tannin), and 1 h boil water strength (866% improvement, from 0.06 to 0.52 MPa). As preheating passed from 40 °C to 60 °C and then 80 °C, the shear strength of the bonded plywood progressively decreased. This is of interest as it means that if the pretreating is done at higher temperature, the reaction has progressed far too much to be effective when heating plywood in the hot press. The indication then is that the viscosity and diffusion hindrance of the reaction product progresses so much before application on the plywood veneers to cause both substrate wetting and underpenetration problems; in short, the material may well be already progressively too cross-linked, and hence inactive when arriving in the hot press. It also means that a moderate reaction temperature of pretreatment, while forming already a pre-polymer between SPI and tannin, does not cause any extreme and inactivating cross-linking, but allows the material formed to wet and adhere well to the wood substrate. This means that the final cross-linking then occurs in the hot press, thus functioning as any thermosetting wood adhesive.

**Figure 2:**
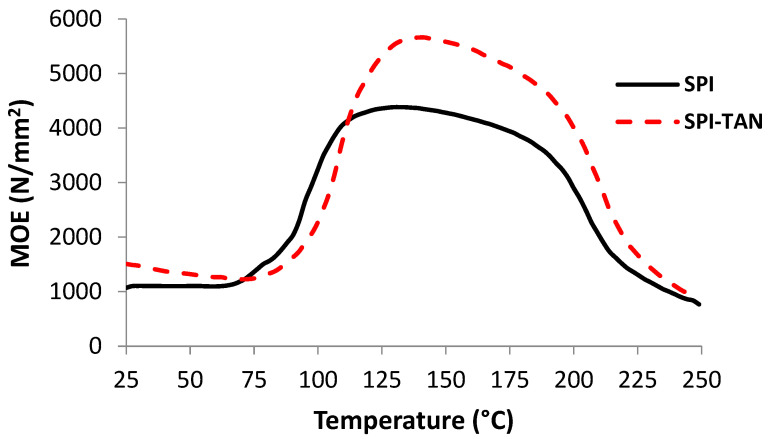
Thermo-mechanical analysis of quebracho tannin–soy protein adhesive. SPI: Soy protein isolate; TAN: Tannin.

**Figure 3:**
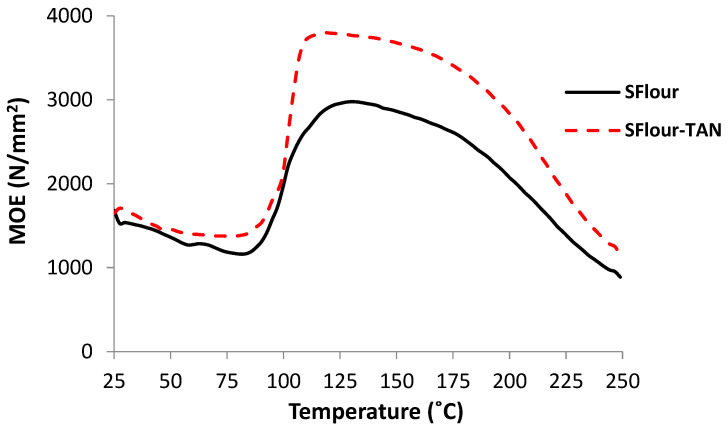
Thermo-mechanical analysis of quebracho tannin–soy flour adhesive. SFlour: Soy flour; TAN: Tannin.

The thermomechanical analyses of SPI–quebracho tannin and soy flour–quebracho tannin in 90:10 dry weight proportions are shown in Figure 2 and Figure 3. The indications are that the presence of even a relatively low percentage of tannin (10%) clearly improves the maximum value of the joint modulus of elasticity (MOE), hence improving joint strength, in both the case of SPI (from 4000 MPa to 6800 MPa, Figure 2) and soy flour (from 2900 MPa to 3800 MPa, Figure 3). The maximum MOE value obtained for the SPI–tannin was markedly higher than that obtained for the soy flour–tannin case. This was expected, as the protein content of soy flour is 50% and it is only the protein in the soy flour that reacts with the tannin; the rest of the flour probably not only did not participate in bond formation, but moreover also increased diffusion hindrance.

**Figure 4:**
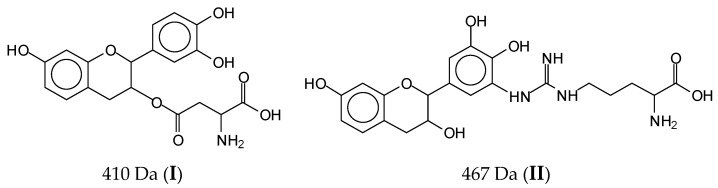
Species identified (**I** and **II**) indicating the two ways of reacting of protein amino acids with flavonoid tannin sites.

**Figure 5:**
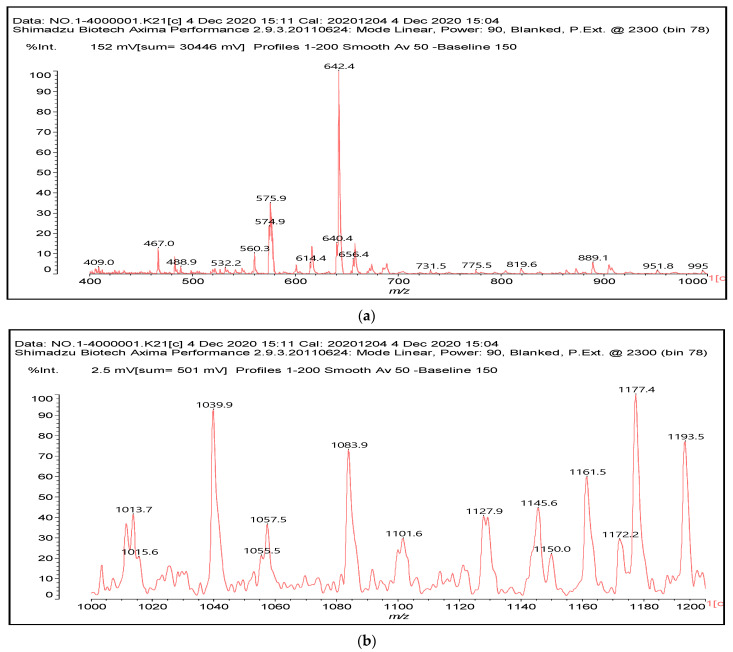

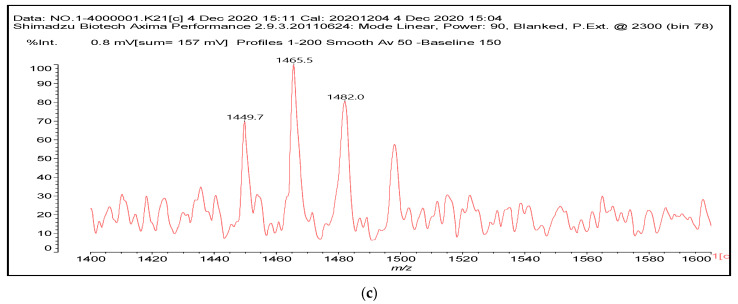
MALDI ToF spectra of SPI–quebracho tannin extract adhesive: (**a**) 400–1000 Da range; (**b)** 1000 Da–1200 Da range; (**c**) 1400–1600 Da range.

MALDI ToF spectrometry gave clear indications of what occurred in the reaction of SPI with condensed flavonoid tannin. It is known that at ambient temperature, only ionic bonds and secondary interactions occur between a tannin and a protein, while covalent bonds progressively form as the temperature increases [23,24]. The only covalent bonds that could form between the protein and a tannin flavonoid unit are those between amino acids side chains presenting a –NH_2_ [27,28,33,34] or a –COOH group. Thus, only arginine, glutamic acid, and aspartic acid are the amino acids in major proportions in soy protein that are capable of participating in reacting covalently the tannin with the protein, and these are the amino acids in major proportion in soy protein [23,24]. The lower reacting amido-groups of the peptide bonds in the main skeletal chain of the protein are most unlikely to react with the tannin. Thus, compounds obtained by the reaction of single amino acids in which the only amino group is the one that will participate in the peptide bond also observed by MALDI ToF must not be considered as regards to the contribution of the tannin cross-linking of the protein. Among the compounds observed by MALDI ToF, the only ones to be considered as contributing to linking and cross-linking the whole protein were the couplings at 410 Da, 467 Da, 483 Da, 575 Da, 601 Da, 688 Da, 889 Da, 1128 Da, 1146 Da, 1161 Da, 1177 Da, and 1194 Da (Figure 5a–c), but some of these appeared to be the ones forming more easily. They could be linked through an ester bond (i.e., 410 Da) or through the substitution of an –NH– for the OH group of a flavonoid unit (the great majority of the others). In the first case, the favorite reaction point is the aliphatic alcoholic –OH at C3 of the flavonoid (**I**), while in the second case the favorite linkage is at any one of the acid phenolic –OH groups (**II**) (Figure 4). 

In Figure 5a–c and in Table S1 (Supplementary Material) are shown the links formed at the higher temperature of 80 °C between tannin monomers and oligomers with both amino acids and short peptide sequences of predominant amino acids present in soy protein. Arginine, glutamic acid, and aspartic acid, in relative proportions of 7.7%, 19.2%, and 11.5% are present, respectively, in soy protein [35], the first due to its –NH_2_ and =NH side chain groups capable of reacting with the acid phenolic –OH of the tannin [24,28,33,34], and the other two due to the presence in each of an extra –COOH side chain group leading to esterification of the aliphatic alcoholic –OH on the C3 site of tannin flavonoids.

**Figure 6:**
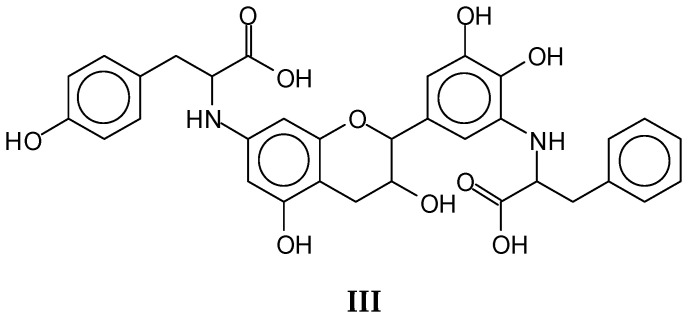
Example of a species (**III**) not contributing to the linking of the protein with the tannin, as the amino group involved in the reaction it cannot react once it is an amido group in the peptide chain.

An example of species not contributing to the cross-linking of the protein by the tannin are the species represented by the peaks at 614–615 Da (with no Na^+^) and 640–642 Da (with Na^+^). The species (**III**) forms, but by reacting with the amino groups that in the protein participate in the peptide bond (Figure 6).

**Figure 7:**
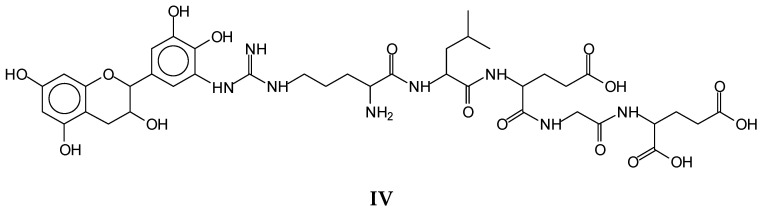
Example of an identified species (**IV**) indicating that cross-linking between a proteinand a tannin occurs. It depicts the species formed by reaction of a gallocatechin reacted with a peptide formed by arginine–leucine–aspartic acid–glycine–glutamic acid.

Conversely, examples of species that contribute to the tannin induced cross-linking of the soy protein at higher temperatures are the species at 889 Da (**IV**), a gallocatechin covalently linked to a peptide formed by arginine–leucine–aspartic acid–glycine–glutamic acid (Figure 7).

**Figure 8:**
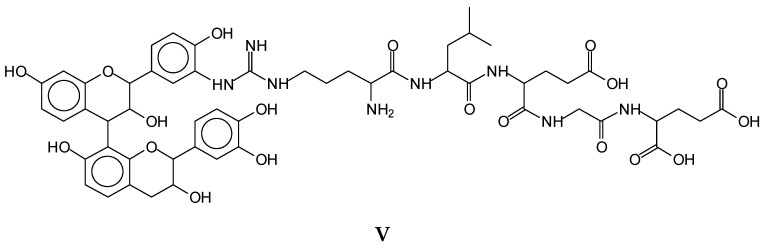
Example of a species (**V**) contributing to protein and tannin cross-linking in which a flavonoid oligomer is shown to react with the side chains of a peptide oligomer chain.

The same peptide fragment is found linked to flavonoid dimers, as for example the species at 1128–1130 Da (**V**), representing a fisetinidin dimer arginine–leucine–aspartic acid–glycine–glutamic acid, and the equal species in which a fisetinidin–catechin dimer, or a robinetinidin–catechin dimer, or a gallocatechin–robinetinidin dimer, or a gallocatechin dimer are linked to the same peptide fragment, respectively, represented by the peaks at 1146 Da, 1161 Da, 1177 Da, and 1194 Da (Figure 8). (Table S1, Supplementary Material).

**Figure 9:**
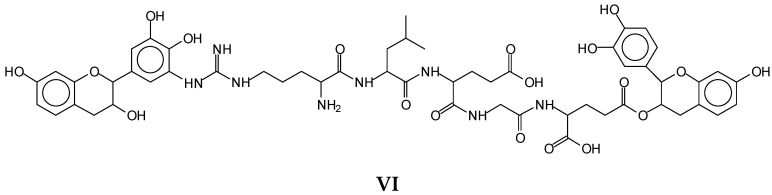
Example of a species (**VI**) of the same molecular weight of species **V** in which the peptide chain oligomer is linked to two flavonoid monomer units of tannin oligomers, showing the possibilities of cross-linking of the adhesive system.

Equally, this series of covalently linked oligomers can be interpreted as two flavonoid units being bridged by the same arginine–leucine–aspartic acid–glycine-glutamic acid peptide as, i.e., for the peak at 1128–1130 Da as in the alternative structure (**VI**)(Figure 9). 

The presence of structures of the same nature as structures **V** and **VI**, and even of higher molecular weight (Table S1, Supplementary Material) indicates that peptide sequences are linked to tannin oligomers, and that peptide sequences can form bridges between flavonoid units, either alone or in flavonoid tannin oligomers. It is equally evident that while at ambient temperature ionic bonds are found [23,24], at 80 °C instead no ionic bond linked products are formed, the totality of the reaction products being covalently bound, meaning that the material starts to be quite advanced in cross-linking, confirming the plywood shear strength results in Figure 1.

## 4. Conclusions

The title of this paper is “a study of concept…” because what disclosed and found in the work presented here does not pretend to be only applicable by itself and not even to be the final word on soy or protein wood adhesives and on wood adhesives from totally bio-sourced natural materials of vegetable origin. It is presented with the clear intention that it will be applicable to many other different approaches by other research groups working on wood adhesives from bio-sourced materials, either already published, or still to be developed.

It shows that pre-reacting just soy protein with a tannin extract at a moderate heat leads to the formation of covalent bonds between the two, final cross-linking being achieved in the hot pressing of wood panels, here of plywood. It shows also that excessive pre-reaction at too high a temperature cross-links prematurely to too high an extent such that the SPI–tannin resin then cannot work anymore as a still reactive thermosetting adhesive during wood panel hot-pressing.

The work shows that an applicable thermosetting wood adhesive can be totally free of any synthetic material by reacting covalently a protein, here a soy protein hydrolysate, with a vegetable tannin extract. Many more improvements can be sought of this initial concept, as to reinforce it with small amounts of adaptive chemicals or biochemicals, as already done on different soy adhesive approaches by several other research groups, opening an alternative means of research of interest.

## Data Availability

The data presented in this study are available upon request from the corresponding authors.

## Supplementary Materials

The following are available online at https://www.mdpi.com/article/10.3390/polym14061150/s1, Table S1: Assignments of molecular structures from MALDI ToF spectrometry for soy protein isolate (SPI) and Quebracho tannin extract resin prepared at 80 °C.
