# Supplementary material for: A Study of Concept to Prepare Totally Biosourced Wood Adhesives from Only Soy Protein and Tannin
Source: Polymers (Basel). 2022 Mar 13;14(6):1150. doi: 10.3390/polym14061150 (PMC8948876; doi:10.3390/polym14061150)

SUPPLEMENTARY MATERIAL

A STUDY OF CONCEPT TO PREPARE TOTALLY BIO WOOD ADHESIVES FROM ONLY SOY PROTEIN AND TANNIN

Saman Ghahri [1]*, Antonio Pizzi [2]*, Reza Hajihassani [1]

Table S1. Assignments of molecular structures from MALDI ToF spectrometry for Soy Protein Isolate (SPI) and Quebracho tannin extract resin prepared at 80°C.

---

410 Da = fisetinidin-aspartic acid with Na^+

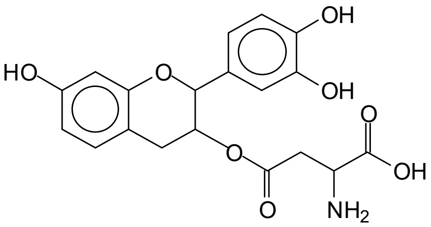

467 Da = robinetinidin-arginine, with Na^+, calculated 469 Da.

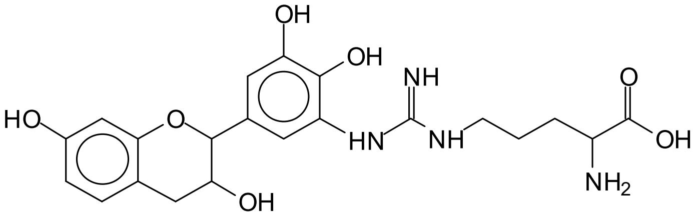

483 Da = delphinidin-arginine with Na + (calculated 485 Da

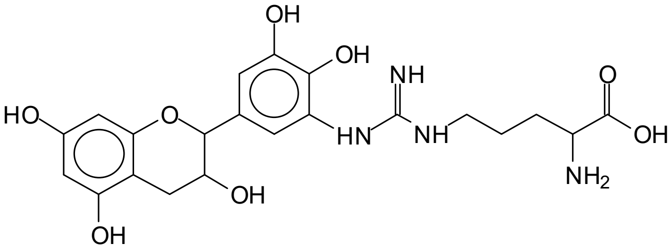

576 Da = aspartic acid-delphinidin-arginine, no Na^+, and 600 Da with Na^+

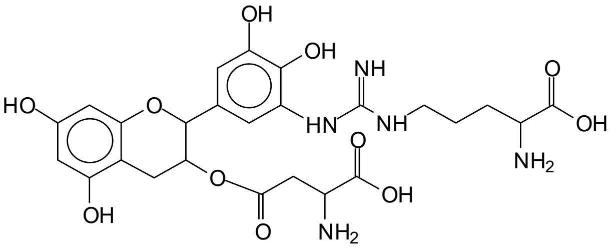

614-616 Da = tyrosine-delphinidin-phenylalanine no Na+, calculated 616

640-642 Da = tyrosine-delphinidin-phenylalanine with Na+, calculated 640 Da

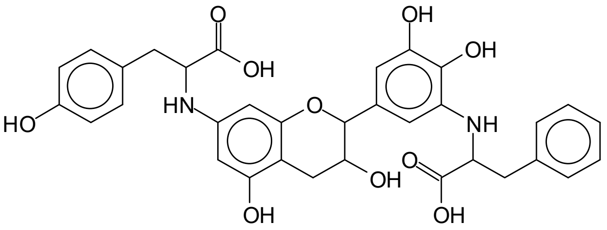

688 Da = no Na+, calculated 688.7 Da., robinetinidin-arginin-leucin-aspartic acid

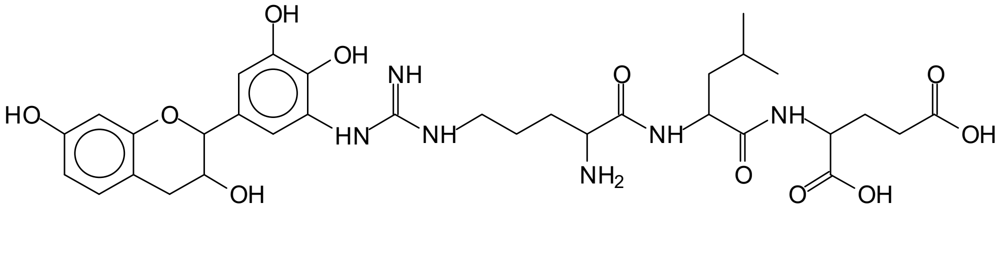

889 Da = no Na+, calculated 890 Da, gallocatechin-arginine-leucin-aspartic acid-glycin-glutamic acid

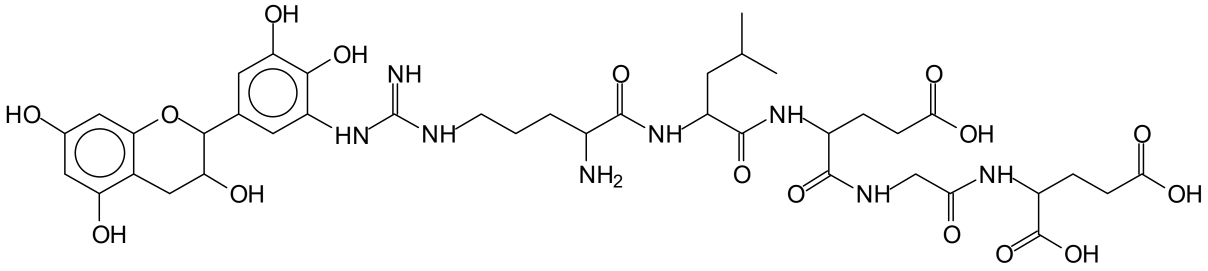

1128-1130 Da = no Na+, fisetinidin dimer-arginine-leucin-aspartic acid-glycin-glutamic acid

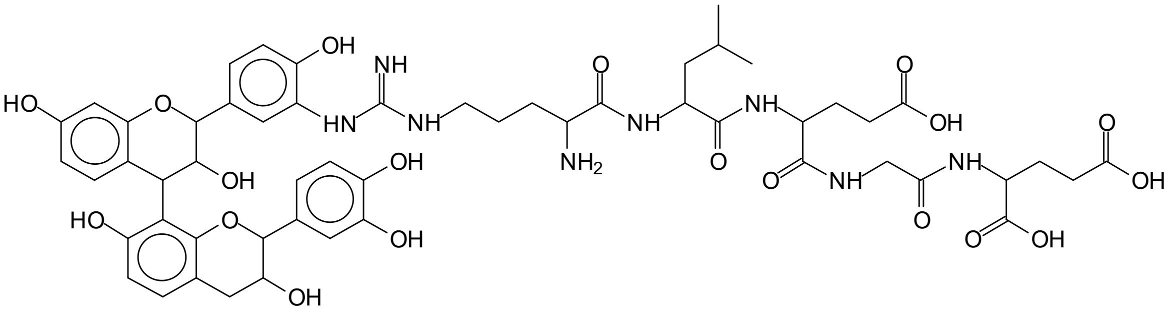

OR

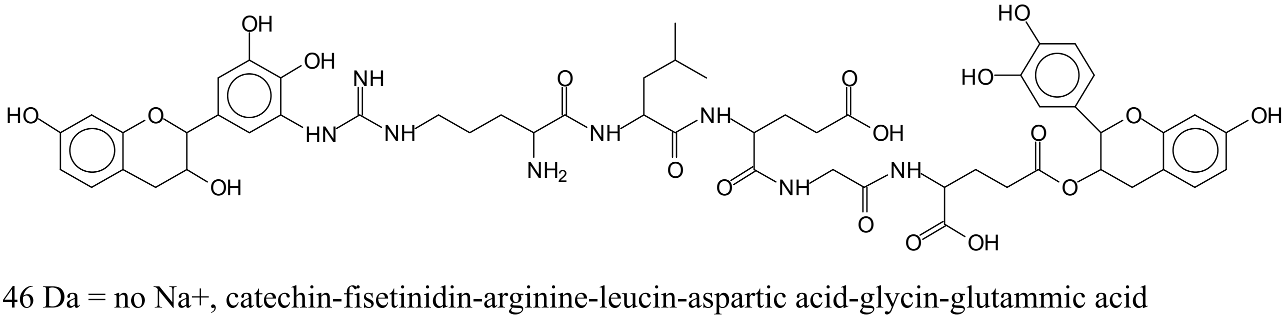

1146 Da = no Na+, catechin-fisetinidin-arginine-leucin-aspartic acid-glycin-glutammic acid

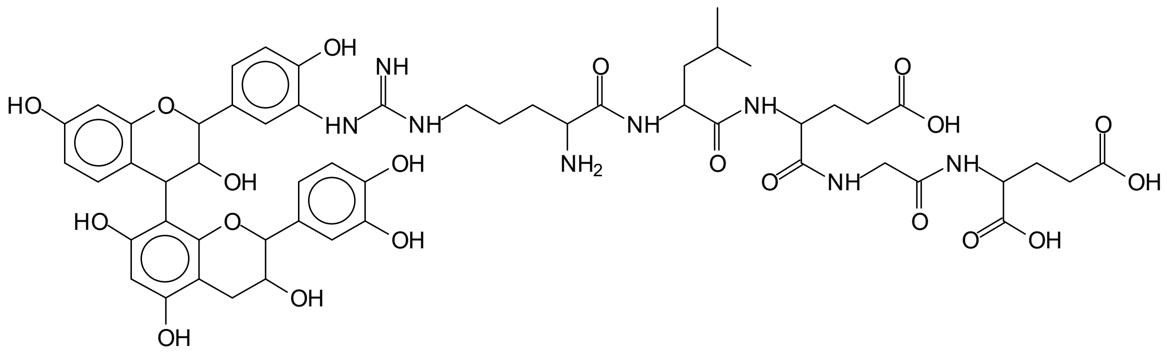

OR

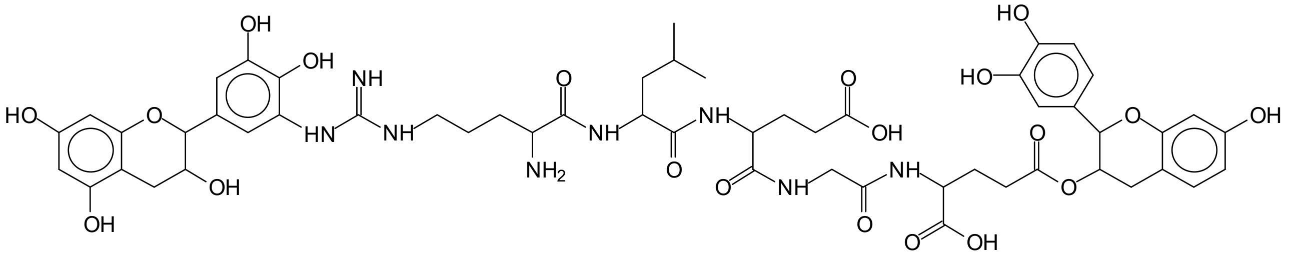

1161 Da = no Na+, calc. 1162 Da, catechin-robinetidin-arginine-leucin-aspartic acid-glycin-glutamic acid

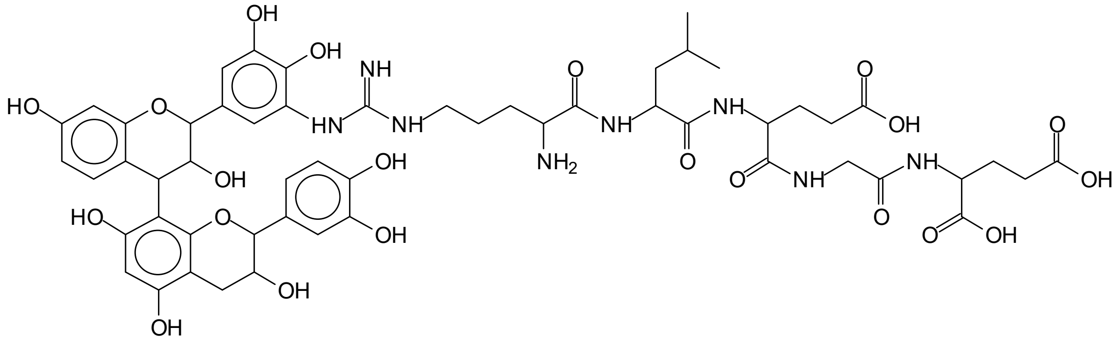

1177 Da = no Na+, calc. 1178 Da, delphinidin-robinetidin-arginine-leucin-aspartic acid-glycin-glutamic acid

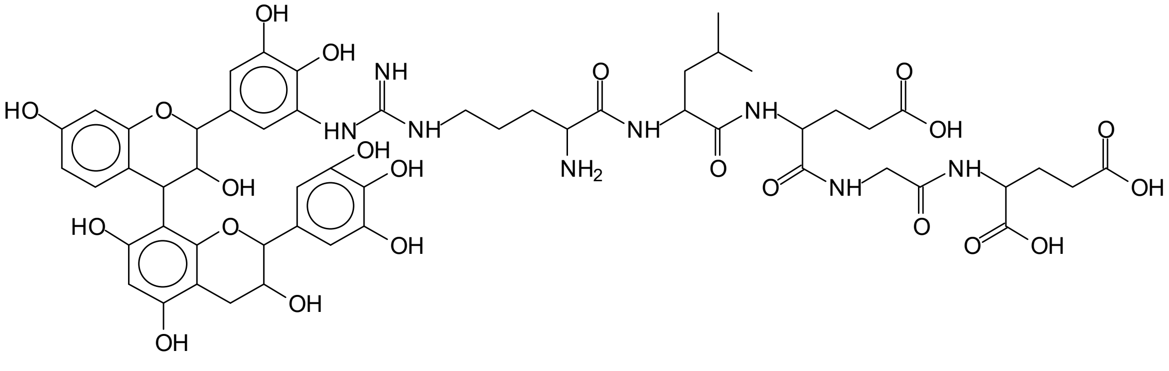

1194 Da = no Na+; calc. 1194 Da, delphinidin dimer-arginine-leucin-aspartic acid-glycin-glutamic acid

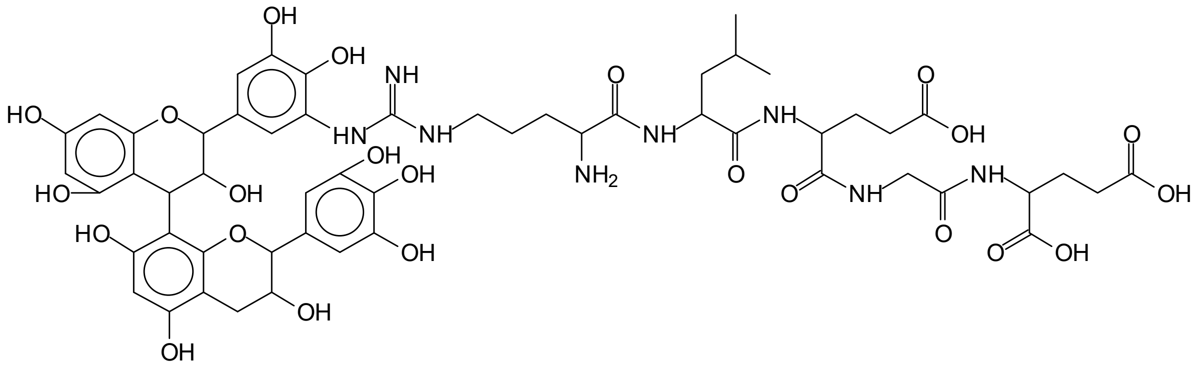

1465 Da = no Na+, glutamic acid-glycine-aspartic acid-robinatenidin-catechin--arginine-leucin-aspartic acid-glycin-glutamic acid

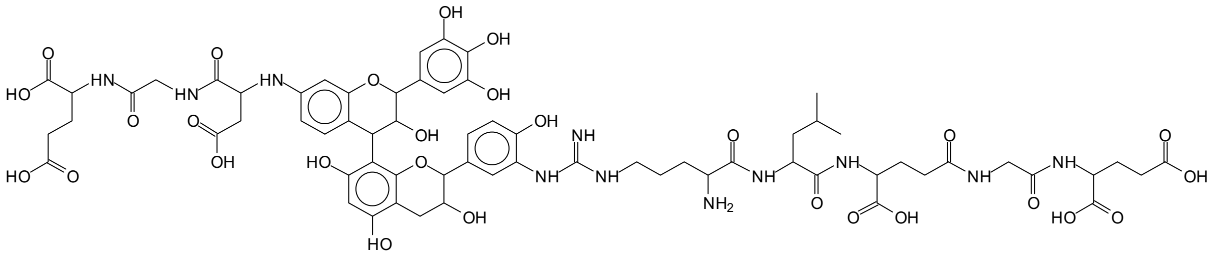

1481 Da = no Na^+, glutamic acid-glycine-aspartic acid-robinatenidin-gallocatechin--arginine-leucin-aspartic acid-glycin-glutammic acid

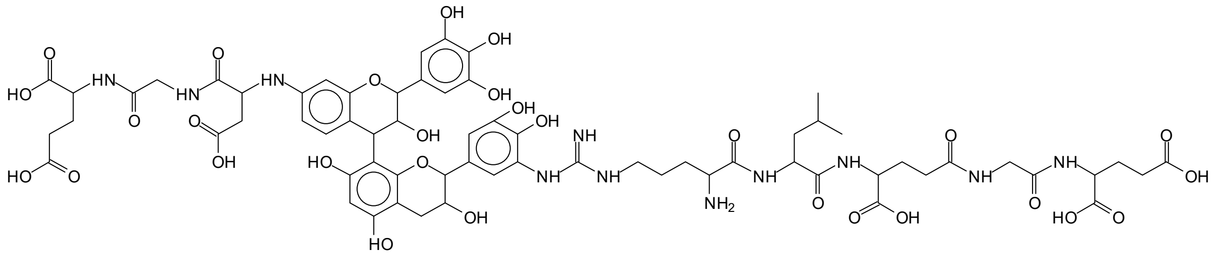

1497 Da = no Na^+, glutamic acid-glycine-aspartic acid-gallocatechin dimer-arginine-leucin-aspartic acid-glycin-glutammic acid

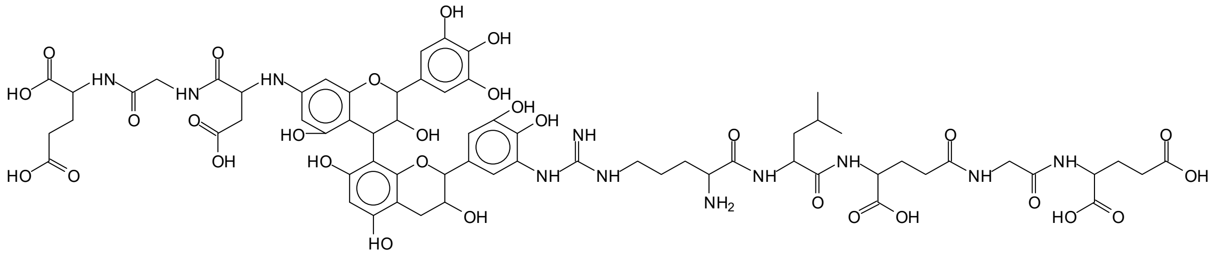

Supplement: Supplementary file 1 [file polymers-14-01150-s001.zip › polymers-1627863-supplementary.pdf]
